# Inulin-Modified Liposomes as a Novel Delivery System for Cinnamaldehyde

**DOI:** 10.3390/foods11101467

**Published:** 2022-05-18

**Authors:** Minxing Xue, Jin Wang, Meigui Huang

**Affiliations:** Department of Food Science and Engineering, College of Light Industry and Food Engineering, Nanjing Forestry University, Nanjing 210037, China; minxingxue@njfu.edu.cn (M.X.); wangjin0077@njfu.edu.cn (J.W.)

**Keywords:** liposomes, surface modification, inulin, physical stability

## Abstract

Cinnamaldehyde as an antioxidant was encapsulated in inulin-modified nanoliposomes in order to improve its physical and antioxidant stability. The microstructure, particle size and volume distribution of cinnamaldehyde liposomes were characterized by atomic force microscopy (AFM) and dynamic light scattering (DLS). The particle size and polydispersion index (PDI) values of the inulin modified liposomes were 72.52 ± 0.71 nm and 0.223 ± 0.031, respectively. The results showed that the liposomes after surface modification with inulin remained spherical. Raman and Fourier transform infrared (FTIR) spectra analysis showed that hydrogen bonds were formed between the inulin and the liposome membrane. Inulin binding also restricted the freedom of movement of lipid molecules and enhanced the order of the hydrophobic core of the membrane and the polar headgroup region in lipid molecules. Therefore, the addition of different concentrations of inulin influenced the permeability of the liposome bilayer membrane. However, when inulin was excessive, the capacity of the bilayer membrane to load the cinnamaldehyde was reduced, and the stability of the system was reduced. Additionally, the encapsulation efficiency (EE) and retention rate (RR) of cinnamaldehyde from inulin-modified liposomes during storage were determined. The EE value of the inulin modified liposomes was 70.71 ± 0.53%. The liposomes with 1.5% inulin concentration had the highest retention rate (RR) and the smallest particle size during storage at 4 °C. The addition of inulin also enhanced the thermal stability of the liposomes. Based on the results, the surface modification improved the oxidation stability of liposomes, especially the DPPH scavenging ability. In conclusion, these results might help to develop inulin as a potential candidate for the effective modification of the surface of liposomes and provide data and conclusions for it.

## 1. Introduction

Essential oils have been studied for more than 60 years, but more interest has emerged in the last decade due to their wider pharmaceutical and therapeutic uses [1]. The most investigated properties are their antioxidant, anti-inflammatory, antibacterial, anti-anxiety and wound healing activity. Cinnamaldehyde, the most abundant component of the cinnamon essential oil, is extracted from the bark, leaves, roots and flowers of cinnamon plants (genus Cinnamomum). Cinnamaldehyde exhibits potential antioxidant, anti-bacterial, anti-diabetic, anti-obesity and anti-cancer functions [2]. Moreover, cinnamaldehyde has been approved by the Joint FAO/WHO Expert Committee on Food Additives (JECFA) for the potential application as a food-flavoring agent [3]. However, the major limiting factors affecting its applications include hydrophobicity, high volatility and oxygen and temperature sensitivity. An effective strategy to overcome these issues in delivery systems is encapsulation. Liposome-based encapsulation systems represent a feasible and efficient approach to modulate the sustainable release of essential oils, improve their physical stability and bioactivity, protect them from interactions with the environment and reduce their toxicity and volatility [4,5].

Liposomes as spherical vesicles are composed of dispersing amphiphilic lipids with hydrophilic heads and hydrophobic tails in an aqueous phase. Liposomes have the advantages of lowering biological toxicity, a high drug loading rate and relative stability in vivo and have been regarded as an ideal drug delivery system for both hydrophilic and hydrophobic functional compounds [6]. Although liposome encapsulation can prolong the activity of bioactive agents during storage, the practical applicability of liposomes is impeded by many barriers, especially in the oral delivery systems, such as instability in the gastrointestinal tract, difficulties in crossing biomembranes and mass production problems. Additionally, during food processing operations, liposomes may be exposed to high temperature and ultraviolet light, which may alter their membranes. Liposome properties differ considerably with lipid composition, surface charge, size and the method of preparation. By modulating the compositions of the lipid bilayers and adding polymers or ligands, both the stability and permeability of liposomes can be greatly improved for oral drug delivery. Generally unmodified liposomes are more prone to aggregation, fusion and oxidation, thereby destroying the integrity of liposomes and causing the leakage of encapsulated substances [7]. To date, liposomes have been broadly catagorized into many different “generations”. For instance, the first generation of liposomes (conventional liposomes without any structural modification) is mainly composed of neutral and/or negatively charged phospholipids and cholesterol. Nevertheless, they have been associated with many issues such as low loading efficiency, easy drug or bioactive compound leakage and so on. Accordingly, the second generation of liposomes is based on the surface modification with inert polymeric molecules, such as polysaccharides, oligosaccharides, synthetic polymers and glycoproteins [8]. Due to the surface modification of liposomes, the second-generation liposomes are more suitable for protecting bioactive agents from degradation. They can improve the controlling release properties and enhance environmental stability. A number of studies have shown that liposomes were used to encapsulate antimicrobials, antioxidants, proteins, peptides, enzymes, fatty acids, flavors and essential oils [9]. Moreover, a variety of substances can be used to form a protective coating, such as chitosan, peptide, pectin, alginate and so on [10,11,12,13,14].

As a result, many researchers have attempted to deposit biopolymers onto the surface of liposomes in order to preserve their structure and increase their kinetic and mechanical stability [10]. Previous studies have found that cinnamaldehyde liposomes modified by polydiacetylene N-hydroxysuccinimide (PDA-NHS) as antibacterial food packaging are effective antimicrobial agents for *Escherichia coli* W1485 and *Bacillus cereus* ATCC 14579, showing significant anti-microbial activity [15]. Carboxymethyl cellulose (CMC)-modified cinnamaldehyde liposomes displayed higher stability compared to the control; this was because of the steric hindrance among liposome vesicles created by CMC [11]. Pertinently, CMC increased the viscosity of the continuous phase to prevent vesicle aggregation [11]. Furthermore, the physical decoration of chitosan improved the encapsulation efficiency of cinnamaldehyde-loaded liposomes, while chitosan and cinnamaldehyde exerted cumulative and synergistic bacteriostatic effects in liposomes [16].

Inulin polysaccharide is composed of D-fructose units linked with the end of glucose residue by β (2 → 1) linkages [17]. Inulin was approved as Generally Recognized As Safe (GRAS) by the US Food and Drug Administration (FDA) in 2002 [18]. Inulin has excellent biocompatibility and low toxicity; it can be used in pharmaceuticals, cosmetics and the food industry and can combine with liposomes [14,19,20]. Inulin can be used to decorate the surfaces of liposomes without negatively affecting the taste of food. The stability of liposomes has been closely related with their structure. To the best of our knowledge, there have been few studies that have reported on the stability and mechanism of cinnamaldehyde liposomes modified by inulin.

Therefore, this work aimed to investigate the effect of inulin based surface modification on the physicochemical properties of cinnamaldehyde liposomes, including particle size, polydispersity index and encapsulation efficiency. The influence of inulin on phospholipid membrane morphology and fluidity was further clarified through atomic force microscopy, Raman spectroscopy and Fourier transform infrared (FTIR) spectroscopy analysis. In addition, the storage stability, heat resistance and antioxidant properties of liposomes were also evaluated. The results of this study will provide a fundamental understanding on the potential food applications of inulin in modifying liposomes as an efficient delivery system.

## 2. Materials and Methods

### 2.1. Materials

L-A-phosphatidylcholine (from soybean, purity > 90%) was purchased from Shanghai Macklin Biochemical Co., Ltd. (Shanghai, China). Cinnamaldehyde (purity ≥ 95%) and inulin (from chicory, purity > 90%) was purchased from Shanghai Aladdin Biochemical Technology Co., Ltd. (Shanghai, China). 2,2-Diphenyl-1-picryl-hydrazyl (DPPH) was purchased from Sigma chemical Co. (St Louis, MO, USA). The other chemicals used in the experiments were of analytical grade.

### 2.2. Liposomes Preparation

The thin-film hydration method was applied to prepare cinnamaldehyde-loaded liposomes according to our previous report [21]. Soy lecithin (1 g), Tween 80 (0.2 g) and cinnamaldehyde (0.12 g) were mixed in 5 mL of anhydrous ethanol at room temperature until all solids are completely dissolved. After solubilization, the liposomal system was dried using a rotary evaporator under reduced pressure at 45 °C. Thereafter, the dried thin film was hydrated with 0.01 M phosphate-buffered saline (pH 6.5) at 45 °C under stirring conditions (500 rpm) for 1 h. Subsequently, the liposomal suspensions were sonicated in an ice-cold water bath for 5 min at 300 W under the conditions of a pulse of 5 s on and 5 s off using a probe sonicator (Model 505 Sonic Dismembrator, Thermo Fisher Scientific, Waltham, MA, USA). Inulin was dissolved in the same phosphate buffer solution (0.01 M, pH 6.5). Then, the solution was added dropwise to the bare liposomal suspensions with a magnetic stirrer at 500 rpm for 1 h. In the liposomal suspension system, concentrations of soy lecithin, Tween 80 and cinnamaldehyde were kept at 25 mg/mL, 5 mg/mL and 3 mg/mL, respectively, and the final concentrations of inulin were set as 0, 0.25, 0.5, 1, 2 and 4 mg/mL. Then, the liposome solution was filtered by 0.22 μm membrane. The samples were sealed with nitrogen and stored in headspace vials at 4 °C in the dark until use. The pure liposomes without cinnamaldehyde-loading were used as the blank sample.

### 2.3. Particle Size Distribution Analysis

The particle size and polydispersity index (PDI) of the liposomes were measured with polystyrene cuvettes by the dynamic light scattering (DLS) method using a Zetasizer analytical instrument (Malvern Zetasizer Nano ZS90, Malvern Instruments Ltd., Malvern, UK) with a He/Ne laser (λ = 633 nm) and a scattering angle of 90° and performed at 25 °C. In order to eliminate multiple scattering phenomena, all samples were diluted 10 times with the phosphate buffer before analysis. The average particle diameter and PDI were calculated from 14 runs for each record, and each sample was analyzed at least three times.

### 2.4. Encapsulation Efficiency (EE) and Retention Rate (RR)

Determination of free cinnamaldehyde content: The content of free cinnamaldehyde was determined by the petroleum ether washing method [22]. Briefly, 1 mL of liposomes was mixed with 3 mL petroleum ether and vortexed for 2 min at 25 °C. The resulting solution was centrifuged at 3000 rpm for 5 min, and the supernatant was collected. After repeating this process once, the resulting solution was purged of petroleum ether with nitrogen, and then ethanol was added, the volume being made up to 10 mL. The absorbance was measured using a spectrophotometer (UVmini-1240, Shimadzu instrument Corp., Kyoto, Japan) at 286 nm, with the control sample as a blank.

Determination of total cinnamaldehyde content: The measurement was carried out according to the method described by Wang [22], with a slight modification. Briefly, 0.5 mL of liposomes was mixed with 1 mL Tween 80-ethanol solution (10%, *w*/*w*) and vortexed for 1 min at 25 °C. Thereafter, the obtained solution was diluted to 10 mL with deionized water. The control sample performed in the same manner was used as the blank, and the absorbance was measured at 286 nm.

Each experiment was carried out in triplicate. The encapsulation efficiency (EE) and retention rate (RR) were determined as follows:

$$\mathrm{EE}\;(\%)=\frac{\mathrm{total}\;\mathrm{cinnamaldehyde}-\mathrm{free}\;\mathrm{cinnamaldehyde}}{\mathrm{total}\;\mathrm{cinnamaldehyde}}\times 100$$

$$\mathrm{RR}\;(\%)=\frac{\mathrm{Encapsulated}\;\mathrm{amount}\;\mathrm{of}\;\mathrm{cinnamaldehyde}\;\mathrm{after}\;\mathrm{storage}}{\mathrm{Encapsulated}\;\mathrm{amount}\;\mathrm{of}\;\mathrm{cinnamaldehyde}\;\mathrm{initially}\;\mathrm{prepared}}\times 100$$

### 2.5. Storage and Thermal Stability of Liposomal Formulations

The samples were stored under N_2_ atmosphere at 4 °C for 40 days in order to evaluate the storage stability of the liposomes. The samples were taken out at regular intervals to measure the retention rate (RR), particle size and PDI. The experiments were performed in the same manner as described above.

For the determination of temperature stability, the liposomes were incubated in a shaking water bath under different conditions of temperature and time (37 °C, 6 h; 65 °C, 30 min; 90 °C, 1 min).

### 2.6. Atomic Force Microscopy (AFM)

The microstructure of the cinnamaldehyde liposomes was analyzed by an AFM (Dimesion Edge, Bruker, Karlsruhe, German). The liposome suspension was diluted 3000 times with deionized water before analysis. The diluted sample (10 μL) was deposited on the mica sheet and left at room temperature for 3 h. Thereafter, the mica flakes were washed with deionized water to remove salts and non-adsorbed vesicles and then dried in an oven at 25 °C overnight. The images were acquired in Scan Asyst mode with a resolution of 512 to 512 pixels in the dimension of 512 × 512 μm^2^ and analyzed with NanoScope^TM^ Analysis 2.0 software.

### 2.7. Fourier Transform Infrared (FTIR) Spectroscopy Analysis

The attenuated total reflectance (ATR)-FTIR spectra were collected through an IR Affinity-1S spectrometer equipped with a single-reflection ATR accessory (VERTEX 80V, Shimadzu instrument Corp., Kyoto, Japan). The lyophilized liposomes were directly placed on the surface of the ZnSe-ATR crystal. The scanning range was 500–4500 cm^−1^. Each spectrum was scanned 32 times.

### 2.8. Raman Spectra Analysis

Raman spectra of all liposome samples were obtained by a DXR Raman microscope (DXR532, Thermo Fisher Scientific, Waltham, MA, USA) equipped with a 780 nm excitation laser and a 10× objective at room temperature. The resulting laser spot diameter was about 3.0 μm, with a spectral resolution of 5 cm^−1^. The Raman measurements were performed with 24 mW, a 50 μm slit aperture and a 2 s integration time. The ordinary Raman spectrum was baseline corrected, and the Raman intensities (I) were measured as the peak height.

S_L_ refers to the relative intensity of the 2880 cm^−1^ Raman band, which is related to the vibrational coupling between the adjacent chains and gives a semiquantitative measurement of the lateral interactions between the acyl chains. On the contrary, S_T_ refers to the relative intensity of the 1130 cm^−1^ Raman band, which is related to the average number of “trans” bonds in the acyl chain and gives a measurement of the order due to the intrachain structure. The parameters S_L_ and S_T_ were calculated as follows [23]:

S_L_ = (I_CH2_ − 0.7)/1.5, I_CH2_ = I_2890_/I_2850_

S_T_ = I_C=C_/1.77, I_C=C_ = I_1130_/I_1090_

where I_2890_/I_2850_ is the height ratio of the peaks at 2890 cm^−1^ and 2850 cm^−1^, and I_1130_/I_1090_ is the height ratio of the peaks at 1130 cm^−1^ and 1090 cm^−1^.

### 2.9. DPPH Radical Scavenging Assay

The DPPH radical-scavenging activity of the liposomes was measured according to our previous method, with a slight modification [21]. Brielfly, 1 mL of liposomes was mixed with 1 mL of DPPH solution (0.4 mM in ethanol) and then incubated for 40 min at 37 °C in the dark. Instead of the liposomes, the deionized water was mixed with DPPH solution to prepare the control. Instead of DPPH solution, the ethanol was mixed with liposome solution to prepare the blank sample. After incubation, the absorbance of all of the solutions was determined at 525 nm using a Thermo Scientific Microplate Reader (SpectraMax i3X, Thermo Fisher Scientific, Waltham, USA). The calculation of DPPH scavenging capacity was performed by the following equation:

$$\mathrm{DPPH}\;\mathrm{scavenging}\;(\%)=\left(1-\frac{\mathrm{Asample}-\mathrm{Acontrol}}{\mathrm{Ablank}}\right)\times 100$$

### 2.10. Ferric Reducing Power Assay

All of the samples, including the mixtures of cinnamaldehyde-DMSO solution and inulin modified liposomes (1 mL), were added with 1 mL of potassium ferricyanide (2.5%, *w*/*v*) and then incubated at 50 °C for 20 min. After rapid cooling in an ice bath, 5 mL of trichloroacetic acid (10%, *w*/*v*) was added to all of the samples and centrifuged at 3000 rpm for 10 min. Around 2 mL of the obtained supernatant was mixed with 2 mL of deionized water and 0.5 mL of FeCl_3_ (0.1%, *w*/*v*) and incubated at room temperature for 10 min. The absorbance of the solution was measured at 700 nm (A_700nm_) using a Microplate Reader. The determination of reducing power is based on the amount of Fe_4_(Fe(CN)_6_)_3_ production as the index. Antioxidants can reduce K_3_ [Fe(CN)_6_] and then use ferrous ions to produce Fe_4_ [Fe(CN)_6_]_3_, which has a large absorption peak at 700 nm. The higher the absorbance, the higher the reducing power became.

### 2.11. Lipid Peroxidation Measurements

Fatty acid peroxidation product malondialdehyde (MDA) reacted with thiobarbituric acid (TBA) to form a colored complex with a maximum absorbance at 535 nm. According to the method of Tan et al. [24], the concentration of MDA was determined by the spectrophotometry of the TBA reaction. Briefly, 1 mL of liposome sample was added into 5 mL of a solution containing TBA (15%, *w*/*v*), trichloroacetic acid (0.37%, *w*/*v*) and hydrochloric acid (1.8%, *v*/*v*). After mixing, the solution was heated at 100 °C for 30 min. Subsequently, the mixture was quickly cooled in an ice bath, and added a solution containing TBA (15%, *w*/*v*), trichloroacetic acid (0.37%, *w*/*v*) and hydrochloric acid (1.8%, *w*/*v*) to 10 mL. The mixture was centrifuged at 2500 rpm for 5 min, and the supernatant was collected by filtration. The absorbance of the supernatant was measured at 535 nm (A_535nm_) by the Microplate Reader. The MDA concentration was calculated by the following equation:

$$\mathrm{MDA}\;(\mathrm{ng}/\mathrm{mL})=\frac{A_{535\mathrm{nm}}\times 4.15\times 1000}{W}$$

where 4.15 is the conversion rate of milliliter liposomes containing MDA (μg/mL), and W is the lipid content per volume (mg/mL).

The change in thiobarbituric acid-reactive substances (TBARS) was calculated by the following equation:

(1) $$\mathrm{Change}\;\mathrm{in}\;\mathrm{TBARS}\;(\%)=\frac{\mathrm{Ac}-\mathrm{As}}{\mathrm{Ac}}\times 100$$

where As and Ac were the absorbance of inulin-modified liposomes and control liposomes (without modification), respectively.

### 2.12. Statistical Analysis

The data are expressed as a mean value with the standard deviation indicated (mean ± SDI). The experiments were performed in triplicate. Statistical analysis was determined using one-way analysis of variance (ANOVA). Differences were considered significant at *p* < 0.05. All statistical analyses were conducted using the SPSS software (version 14.0; SPSS).

## 3. Results and Discussion

### 3.1. Characterization of Inulin Modified Liposomes

**Figure 1:**
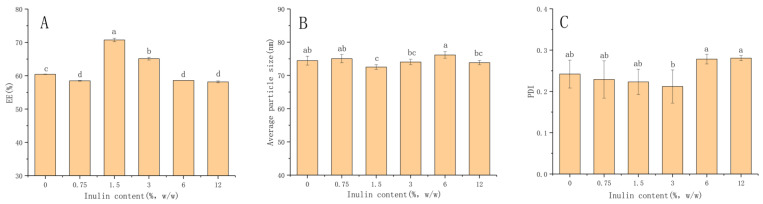
Modified cinnamaldehyde liposomes at different inulin concentrations. (**A**) EE; (**B**) Average particle size; (**C**) PDI. Results are represented as the mean ± sd (n = 3). Different letters represent a significant difference (*p* < 0.05). EE: encapsulation efficiency; PDI: polydispersion index.

The increase in the concentration of antioxidant might induce the pro-oxidation of lipids, leading to the instability of liposomes [25]. So, in the present study, the ratio of cinnamaldehyde to excipients was chosen to be 1:10 (cinnamaldehyde/excipient, *w*/*w*), at which point the encapsulation efficiency and storage stability of liposomes were relatively high. The presence of Tween 80 could induce steric stability and higher flexibility in the lipid bilayer [26]. The tolerance of Tween 80 recommended by FAO/WHO as a food emulsifier is only 0.1–1 (g/100 g) [27]. Therefore, an appropriate ratio of cinnamaldehyde/emulsifier could be used to prepare the liposomes. In the present study, five different concentrations of inulin were tested to modify the liposomes. Inulin was subsequently added after the liposome preparation in order to avoid direct interaction with cinnamaldehyde (located in the hydrophobic core). The inulin solution was added in a dropwise manner to the bare liposome suspensions with magnetic stirring so that the inulin could have sufficient time to contact, react and adsorb on the surface of the liposomes. The EE, average particle size and PDI of liposomes as a function of the inulin concentrations at the initial stage of preparation are shown in Figure 1. The changes of liposomes interacting with inulin had been analyzed using DLS. The average diameter of the liposomes without inulin was 74.45 ± 1.33 nm, while the PDI was 0.242 ± 0.03. The concentration of inulin ranged from 0 to 12%, and the average particle size of all liposome samples was between 70 and 80 nm. The results showed that inulin was not tightly piled up on the surface of the liposomes, as the particle size of the liposomes did not change significantly. It is significant to analyze the changes in liposome particle size during storage afterwards. The EE reflected the loading capacity for the core material cinnamaldehyde of the lipid membranes. It also should be pointed out that the incorporation of cinnamaldehyde had a great impact on the fluidity properties of liposome membranes [16], and inulin affected the cinnamaldehyde encapsulation by affecting the hydrophobicity of phospholipid molecules. As the concentration of inulin increased, EE increased significantly and reached the optimum level. However, further increases in inulin decreased the EE. From the results, EE increased to the highest level of 70.7% when the inulin concentration reached 1.5%. The PDI of all samples was less than 0.3, which indicated the homogenous distribution of inulin on the surfaces of the liposomes.

**Figure 2:**
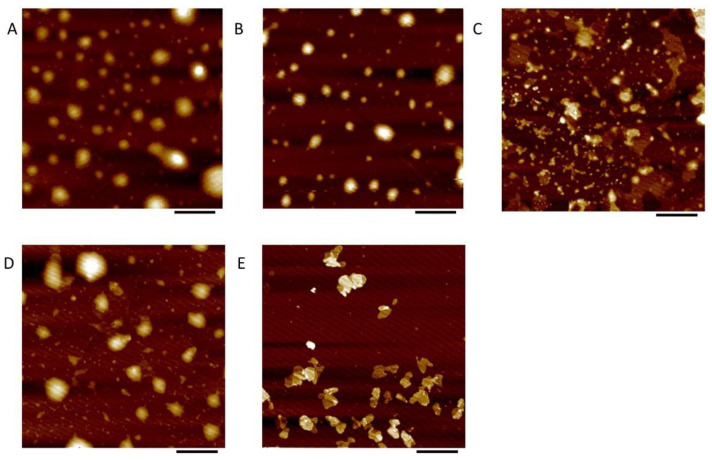
Atomic force microscopy (AFM) images of cinnamaldehyde-loaded liposomes ((**A**–**E**): The inulin concentrations were 0%, 0.75%, 1.5%, 3% and 6%, *w*/*w*, respectively). The scale bars in the figure represent 1 μm.

The morphology and size distribution of the liposomes were observed by AFM (Figure 2). It was found that most of the particles were spherical shaped in the sample without inulin. This illustrated the different morphological behavior in comparison with the inulin modified liposomes. On the other hand, the inulin was well dispersed, without obvious aggregation onto the liposome surface. This indicated that the inulin modification did not produce any significant change in the morphology of the liposome particles.

Compared with the control sample (without inulin) [A], a slight change was observed in the inulin-modified liposomes. At 0.75% (B) and 1.5% (C) concentration, the liposome vesicles maintained a spherical shape, and a similar diameter was obtained compared to that shown in the control sample (A). For the 3% inulin-modified liposomes (D), the liposomes began to aggregate spontaneously. Liposomes with irregular particle shapes appeared in the case of high inulin concentration. This might be due to the formation of inulin crystals after the drying of mica sheets. It indicated that some modified vesicles were in an unstable state during sample preparation. This might be due to the high sensitivity to aggregation, coalescence or particle collapse. However, most of the particles of the inulin coating were spherical, and the surface was smooth, indicating that the inulin modification was evenly distributed on the membrane surface and did not affect the shape of the liposome vesicles. The average liposome diameter derived from the AFM image supported the results of the DLS analysis. However, the height of all of the liposomes was much smaller than their average diameter. This could be due to the collapse of the droplets during the evaporation process and the interaction between the adsorbed liposomes and the supporting surface after their deposition on the mica surface [28]. The effect of inulin modification on the size of the liposomes was associated with their position and orientation in the lipid membrane. The results indicated that the inulin chains might cover the surface of liposomes.

### 3.2. Fourier Transform Infrared (FTIR) Spectroscopy Analysis

**Figure 3:**
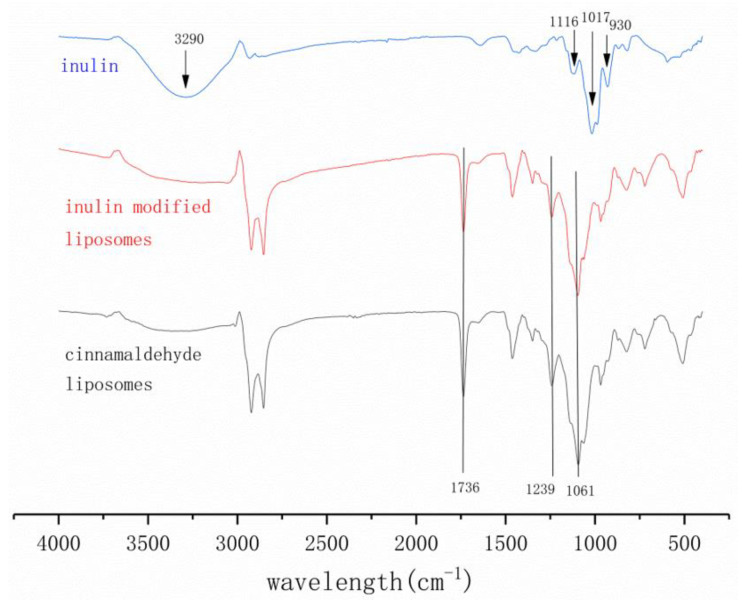
Fourier transform infrared (FTIR) spectra of inulin, inulin modified liposomes and cinnamaldehyde liposomes.

As shown in Figure 3, the interaction between inulin and liposomes was explored by measuring the infrared spectra of inulin, cinnamaldehyde liposomes and inulin-modified cinnamaldehyde liposomes. The spectrum of inulin showed characteristic peaks at 930, 1017 and 3290 cm^−1^. Additional polysaccharide characteristics resulting from the skeletal vibrations of fructose and glucopyranose rings could be observed around 800–1200 cm^−1^. The peaks at 862 and 805 cm^−1^ are due to the CH out-of-plane deformation of inulin, and the peak at 930 cm^−1^ is due to the C–O–C stretching of the fructan ring of inulin. Meanwhile, the absorbance around 600–800 cm^−1^ reflects the absorption of C–H aliphatic bending. Additionally, the peaks located at 1116 cm^−1^ are due to the C–O–H and glycosidic bonds of inulin, and the peaks at 1017 cm^−1^ are due to the CH_2_ stretching and bending vibrations of inulin [29,30]. The peaks at 3290 cm^−1^ are related to hydrogen bonding with hydroxyl groups, and they are reported to be sensitive to water levels, as higher levels of inulin crystallization lead to water induction in the system [31]. Due to dispersion in the liposome system, the inulin concentration decreased and the peak disappeared. The absorption peaks of the cinnamaldehyde liposomes and inulin modified liposomes at 1736 cm^−1^ (C=O), 1239 cm^−1^ (P=O) and 1061 cm^−1^ (P–O–C) correspond to the characteristic groups of phospholipid [27]. Meanwhile, the absorbtion peaks of 1080 cm^−1^ and 1157 cm^−1^ (PO^2−^ symmetric and asymmetric stretching vibrations) were also regarded as characteristic groups of phospholipids [32]. The FTIR spectra of the cinnamaldehyde liposomes and inulin modified liposomes did not show visible differences, indicating that there was no chemical interaction between the inulin and liposomes. The characteristic peak of inulin disappeared at 3290 cm^−1^. This also indicated that inulin might be physically adsorbed on the surface of the liposomes. It is suggested that the P=O bond of the phospholipid in the liposomes might interact with inulin to a certain extent. Such changes might be caused by the hydrogen bonding between the inulin and phospholipid [33]. The shift of the P=O vibration in the spectral position in the FTIR spectra indicated that there was H-bonding. This observation was similar to other reports that inulin might form hydrogen bonds with liposomes [14,34]. The spectral width of the liposomes decreased after being modified with inulin, indicating that the fluidity of the modified membrane was reduced and the stability of the modified liposomes was enhanced [10]. Generally, the negatively charged inulin molecules and negatively charged liposomes display electrostatic repulsion. However, in this study, it could not be precluded that the inulin molecules and the negatively charged liposomes shared partial binding or weak interaction. These results also indicated that inulin had been deposited on the surface of the liposomes, which was consistent with the results obtained by DLS and AFM. There were absorption peaks of 2922 cm^−1^ and 2853 cm^−1^ in both the cinnamaldehyde liposomes and inulin modified liposomes. Those peaks correspond to the stretching vibrations of the C–H bonds (CH_2_ symmetric and asymmetric stretching vibration) [10]. This was consistent with the results of the Raman spectra.

### 3.3. Storage Stability Evaluation

**Figure 4:**
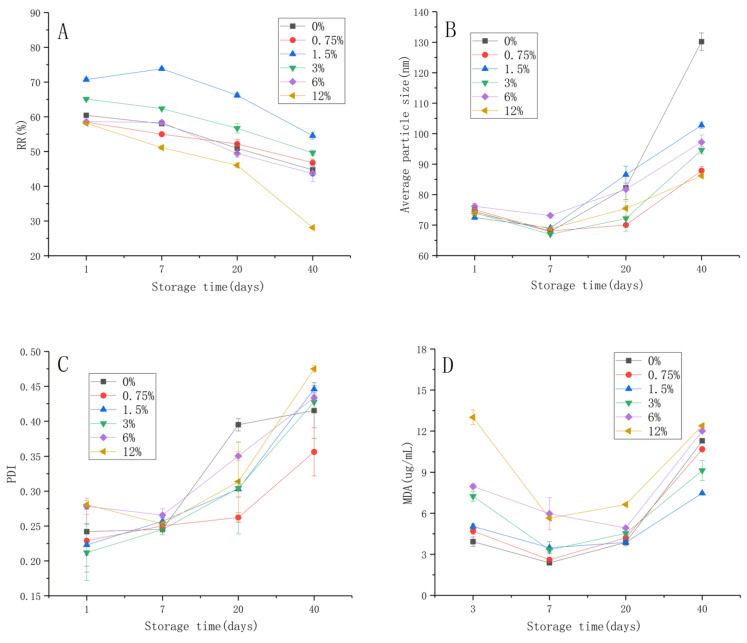
(**A**) Changes in the RR; (**B**) Particle size; (**C**) PDI; (**D**) The content of MDA (MDA value) of modified cinnamaldehyde liposomes with different inulin concentrations of storage. Results are represented as the mean ± sd (n = 3). RR: retention rate; PDI: polydispersion index; MDA: malondialdehyde.

In order to assess the shelf life of liposome samples, the retention rate (RR) during storage at 4 °C was determined. Storage and thermal stability were also expressed as a function of the retention rate of cinnamaldehyde liposomes and the storage time. As shown in Figure 4A, the retention rate of the cinnamaldehyde loaded liposomes, irrespective of different inulin concentrations, decreased with time at 4 °C during storage. The stability of the unmodified liposomes was poor, and the retention rate after 40 days was 44.73%. From the results, the liposomes modified with inulin improved the stability of cinnamaldehyde. The highest stability was achieved in the liposomes modified with inulin concentrations of 1.5% and 3%. The retentions after 40 days reached to 54.55% and 49.63% in the liposomes modified with the inulin concentrations of 1.5 and 3%, respectively. It was found that the liposome added with 12% inulin concentration has decreased stability. This indicated that the high concentration of inulin could reduce the stability of liposomes. Oxidation could change the structure of liposomes, while the modification of inulin could protect the cinnamaldehyde from damage and leakage to varying degrees.

Liposomes are thermodynamically unstable systems. The particles have a high tendency to aggregate and degrade [24]. This will cause the leakage of cinnamaldehyde from the liposomes. As shown in Figure 4B, the particle size of unmodified liposomes increased significantly. For instance, it increased to 130.2 nm after 40 days. However, the particle size of the liposomes modified with inulin remained below 100 nm for 40 days. This was reasonable in comparison with the values of 72.52 nm, 74.03 nm and 76.15 nm found on the first day in the liposomes modified with the inulin concentrations of 1.5%, 3% and 6%, respectively. This result indicated that the addition of inulin inhibited the aggregation of particles and prevented the leakage of cinnamaldehyde. The cinnamaldehyde retention ability of the lipid bilayer was stronger at low inulin concentrations and decreased when the concentration exceeded 6%. The changes in particle size distribution coincided with changes in RR. The RR at the initial stage was a little bit lower than the initial concentration. This indicated that the cinnamaldehyde could not be fully incorporated into the liposome membrane [13]. When inulin was in excess, the capacity of the bilayer membrane to load cinnamaldehyde was reduced, the membrane permeability was enhanced and the stability of the system was reduced. Therefore, the stability of the liposomes first increased and then decreased with the increase of inulin concentration. The ability of cinnamaldehyde to intercalate into liposome membranes is due to its good lipid solubility [2]. The inulin chains interacted with the liposome membrane, inserting into the phospholipid head and reducing membrane fluidity [17]. Meanwhile, the average particle size of all of the samples was similar in the initial stage. This indicated that the modification of inulin did not affect the effect of sonication on liposomes, and the mechanical properties of the membrane therefore remained same [24]. Inulin modification reduced the aggregation of the liposomes and would become a potential pathway to improve liposome stability.

As shown in Figure 4C, the liposome suspension showed inhomogeneity after storage for 40 days (PDI > 0.3). It was found that the particles tend to aggregate/agglomerate at different aggregate sizes. At 20 days, the PDI values of other samples were all below 0.3, except for 6% inulin concentration and the control group, indicating that the system was relatively homogeneous. Due to the presence of polyunsaturated acyl chains in lipid molecules, lipid peroxidation might occur during the preparation process and storage [35]. The secondary product of the peroxidation reaction, MDA, was detected spectrophotometrically in the TBA reaction [24]. The MDA content in the liposome systems during storage was determined, and the results are shown in Figure 4D. From the literature on bilayer membrane peroxidation, there was a consensus that membrane damage was related to the increased membrane permeability in the presence of oxidized lipids [35]. It showed that the addition of different concentrations of inulin would affect the permeability of the lipid bilayer membrane and then affect the leakage of cinnamaldehyde. It was partly due to the different retention of cinnamaldehyde by lipid bilayers at different inulin concentrations. Additionally, it is worth noting that liposomes with 1.5% inulin content had the lowest MDA content, while liposomes with 12% inulin content had the highest MDA content. At the initial stage of storage, cinnamaldehyde reduced the lipid that had peroxidation reaction in the preparation process, and the content of MDA decreased gradually. With the increase in storage time, lipid oxidation would affect membrane permeability, and cinnamaldehyde gradually leaked out. The antioxidant capacity of the whole liposome system was weakened, membrane permeability was improved, lipid peroxidation reaction was intensified and MDA content began to rise. After 40 days of storage, the amount of MDA produced in the liposomes increased considerably; however, the liposome system with the concentration of 1.5% inulin had the strongest antioxidant activity. This was coincidental with the results of RR. The inhibition of lipid peroxidation was due to the effect of inulin on the membrane properties. Inulin was inserted between the lipid headgroups, thereby spacing the acyl chains, and the headgroup was immobilized by the interaction with inulin [36]. Overall, inulin inhibits peroxidation by restricting the movement of lipid molecules to restrict the entry of oxygen into the lipid bilayer.

The thermal stability of liposomes is an important indicator of the efficacy of the drug delivery system. Liposomes are often heated during production or utilization processes, such as pasteurization, high temperature sterilization and in vivo digestion. Elevated temperature disrupted the structure of liposomes and promoted the chemical degradation of cinnamaldehyde [37]. To investigate the temperature resistance of liposomes modified with different concentrations of inulin, the volume distributions of particle size and RR were monitored after heating the samples at desired temperatures. For simulating body temperature, pasteurization and high temperature conditions, the effects of three different heat treatments on the stability of liposomes were studied, viz. low-temperature and long-term heating (37 °C, 6 h), medium-temperature and intermediate-term heating (65 °C, 30 min) and high temperature and short-term heating (90 °C, 1 min).

**Figure 5:**
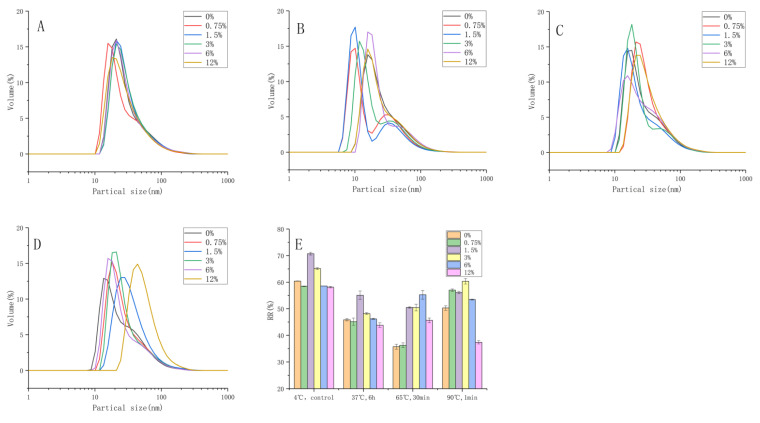
Particle size distribution of liposomes under different storage conditions: (**A**) 4 °C, 2 d (**B**) 37°C, 6 h (**C**) 65°C, 30 min and (**D**) 90°C, 1 min. (**E**) Changes in the RR of different temperatures of storage. The results are represented as the mean ± sd (n = 3). RR: retention rate.

As shown in Figure 5, liposomes with different concentrations of inulin showed a single peak state before heating (A), and the volume distribution was concentrated, with slight differences among the samples. The particle size volume distribution of each liposome sample after incubation at 37 °C for 6 h showed a bimodal shape (B). It showed that, after heating for 6 h, some particles started to aggregate. The concentrations of 0.75%, 1.5% and 3% had a narrow and high volume peak in the range of 50–80 nm, while the peak shapes of 0%, 6% and 12% were relatively low and wide. From the perspective of peak distribution, the particles tended to aggregate and precipitate when the concentration of inulin was too high. However, under the conditions of 65 °C (C) and 90 °C (D), the change in the volume distribution of the particle size was not as obvious as that of 37 °C. Especially at 65 °C (C), it basically still maintained a single peak distribution, but the average particle size became larger. In general, the addition of inulin effectively slowed down the particle aggregation during heating. At 37 °C, the aggregation and adhesion of the liposome vesicles were evident, surely leading to a drop in RR, as shown in Figure 5E, of which 1.5% was significantly higher than in the other groups. The results showed that the presence of inulin enhanced the stability of cinnamaldehyde liposomes against thermal degradation, which might be due to its altered location of cinnamaldehyde in the aqueous phase [37]. Heating resulted in an increase in the average particle size of the liposomes, which was consistent with other findings [37,38,39]. The RR of the liposomes with no inulin modification under all temperature conditions was the lowest, indicating that inulin could effectively protect cinnamaldehyde from leaking when heated. Additionally, pasteurization conditions had minimal effect on the physical properties of the liposomes, which might be due to the ability of inulin to increase the steric and electrostatic repulsion between liposomes, as previously described.

### 3.4. Raman Spectra Analysis

The principle of the Raman spectrum is the inelastic scattering of incident light, and the intensity of the scattered light as a function of the Raman shift produces a spectrum. While in infrared spectroscopy, the interactions between infrared radiation and matter arise from the change in molecular dipoles associated with vibrations and rotations, resulting in spectral characteristics. So, the information provided by FTIR and Raman spectra is complementary [35]. Raman spectroscopy provides strong evidence of the structure of the lipid hydrocarbon chains of liposomes, which include the order parameter changes for the lateral interaction between chains and the longitudinal interactions within the chains, as well as the changes for the trans and gauche conformations of the hydrocarbon chains [10]. Raman spectroscopy has already been used to detect the lipid layered structure of liposomes [40]. The structural changes of the cinnamaldehyde liposomes modified with inulin were observed by the Raman spectra in the present study. The characteristic peaks of the phospholipids in the Raman spectra mainly include the C–H stretch vibration range (2800–3000 cm^−1^) and the C–C stretch vibration range (1000–1200 cm^−1^) [28].

The different structure of the lipid bilayers of the membrane can be studied by observing the ratio of the 2890 cm^−1^ band to the 2850 cm^−1^ band in the Raman spectrum. These two strong bands correspond to the methylene antisymmetric C–H stretch and the methylene symmetric C–H stretch of the acyl chains of lipids, respectively [23]. These two strong bands are very sensitive to the change of their chains and are widely used to study the conformation of lipid bilayers of the membrane. So, S_L_, which is calculated from the ratio of peak intensities of 2890 cm^−1^ to 2850 cm^−1^, is associated with the changes in the lateral interaction between chains. The data of S_L_ and its changing rate are shown in Table 1.

Significant differences can be observed from the intensity ratio of I_2890_/I_2850_ measured at different inulin concentrations. I_CH2_ can be regarded as the ratio of I_disorder_/I_order_, in a simple term. This is complex in polymethylene chains but can be used to observe the structural changes of liquid crystals and lipids. In addition, I_CH2_ is very sensitive to both the molecular environment and conformational disorder [41]. The data show that the I_CH2_ and S_L_ increased with the inulin concentration rising from 0% to 1.5%, and it is indicated that the interchain disorder and lateral packing between the lipid molecules increased. When 0.75% of inulin was added to the cinnamaldehyde liposomes, the I_CH2_ and S_L_ began to rise, and the S_L_ increased by 35.43%, which might indicate that a very small amount of inulin can cause the lateral packing and interchain disorder between lipid molecules increasing. However, S_L_ decreased with a further rise in inulin concentration from 1.5% to 6%. In general, the S_L_ of samples with inulin showed an oscillating trend with the increase of inulin concentration, and they were all higher than the pure liposomes sample, indicating that the presence of inulin can effectively change the fluidity of the bilayer membrane. So, the stability of liposomes can be improved within a certain concentration. In addition, it was found that, with the addition of inulin, the bands of the symmetrical stretching vibration of C–H did not shift (2850 cm^−1^), while the bands of the antisymmetrical stretching vibration of C–H (2890 cm^−1^) basically exhibited a blue shift: 2937 cm^−1^ (0.75%), 2936 cm^−1^ (1.5%), 2929 cm^−1^ (3%) and 2934 cm^−1^ (6%), respectively. So, the addition of inulin did change the microenvironment around the lipid molecular hydrocarbon chains in the bilayers.

The C–C stretch vibration reflects the change of the trans and gauche conformations. The bands at about 1055 cm^−1^ and 1130 cm^−1^ correspond to the all-trans bond stretch vibration of the C–C skeleton, and the band at about 1090 cm^−1^ corresponds to the gauche rotamers. The more all-trans bonds there are, the larger the longitudinal order parameter would be, but it is reversed for the gauche rotamers [42]. These three bands have shifted with different inulin concentrations of cinnamaldehyde liposomes to the following: 1064, 1084, 1126 cm^−1^ (0%); 1064, 1073, 1134 cm^−1^ (0.75%); 1069, 1084, 1129 cm^−1^ (1.5%); 1073, 1080, 1129 cm^−1^ (3%); 1046, 1084, 1129 cm^−1^ (6%); 1080, 1084, 1122 cm^−1^ (12%).

I_C=C_, the intensity ratio of the 1130 cm^−1^ band to the 1090 cm^−1^ band in the Raman spectrum, represents the ratio of the trans conformation to the gauche conformation, which is usually used to reflect the degree of the longitudinal order of liposomes. The higher the intensity ratios I_1130_/I_1090_, the larger the longitudinal order parameter of the liposomes would be. As shown in the Table 1, S_T_ decreased significantly from 1.01 to 0.64 when the inulin concentration increased from 0% to 0.75%. So, the presence of small inulin concentrations might have a significantly disordering effect on the lipid acyl chains of the liposomes. S_T_ increased with further increases in the inulin concentrations until 3%, at which point it decreased. The longitudinal order would be reduced to the lowest level when the concentration of inulin continues to increase to 12%. As the inulin concentration was up to 3%, the longitudinal order parameter was significantly higher than that of the liposomes with no inulin. This means that the gauche conformations decreased, the all-trans bonds increased, the order within the chain increased, the fluidity decreased and the stability of the liposomes increased.

Additionally, the band at 1728 cm^−1^ is attributed to the cis-C=C bond of lipids in the Raman spectrum because of the unsaturated fatty acids which compose lipids of the liposomal bilayers [28].

### 3.5. Antioxidant Activity

**Figure 6:**
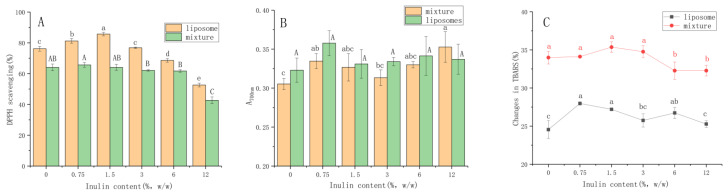
(**A**) DPPH radical scavenging ability; (**B**) Ferric reducing ability (A_700nm_); (**C**) Changes in TBARS of different concentrations of inulin modified cinnamaldehyde liposomes and the mixture of cinnamaldehyde-inulin-DMSO solution and blank liposomes. Results are represented as the mean ± sd (n = 3). Different letters represent a significant difference (*p* < 0.05). DPPH: 2,2-diphenyl-1-picryl-hydrazyl; TBARS: thiobarbituric acid-reactive substances; DMSO: dimethyl sulfoxide.

We simply evaluated the antioxidant activity of cinnamaldehyde liposomes through the in vitro assays of DPPH scavenging, ferric reducing antioxidant power (FRAP) and the inhibition of TBARS formation power. The cinnamaldehyde liposomes had antioxidant properties that could maintain the organoleptic qualities of various foods. The basis of the DPPH method was to reduce DPPH· in an alcohol solution in the presence of antioxidant molecules, thereby capturing the odd electron paired with hydrogen by DPPH [43]. It can be seen from Figure 6A that the mixture of the cinnamaldehyde and blank liposomes also had antioxidant activity, but the scavenging rate of the cinnamaldehyde liposomes after encapsulation was always greater than that of the mixture. The enhanced scavenging activity of the cinnamaldehyde liposomes might be related to the improvement of water dispersion by the encapsulation treatment. In addition, encapsulation also reduced the degree of aggregation of cinnamaldehyde, thereby increasing the antioxidant efficiency [27]. The scavenging rate of the cinnamaldehyde liposomes increased at first and then decreased with the increase in inulin concentration. The appearance of inulin can maintain the original antioxidant properties of liposomes. When the inulin concentration was greater than 6%, the scavenging rate was lower than that of the liposomes that were not modified by inulin. The results showed that the ability to scavenge the DPPH radicals of 1.5% inulin modified liposomes was 85.70%. When the inulin concentration continued to increase to 12%, the scavenging effect decreased to 52.59%. Therefore, the sample with 1.5% inulin concentration showed the highest free radical scavenging activity in the case of an excess of free radicals. This might be related to the altered membrane fluidity of cinnamaldehyde liposomes, and inulin could rigidify the hydrophobic regions of the membrane lipid bilayer [26]. At the same time, an excessively high concentration of inulin would hinder the diffusion of free radicals in space and reduce the reaction of free radicals [36].

The principle of FRAP was to monitor the change of Fe^3+^ to Fe^2+^ caused by electrons in reducing substances [44]. Cinnamaldehyde reduced the Fe^3+^/ferricyanide complex to the form of ferrous by supplying electrons, which had an effective reducing ability. Similar to the DPPH scavenging, the reducing ability of the cinnamaldehyde encapsulated was also improved in comparison with the mixture. Further, it is worth noting that ferricyanide is an oxidant that could hardly pass through the hydrophobic region, so it was widely used to evaluate the position of the redox group in the system [28]. This result indicated that cinnamaldehyde not only distributed at the hydrophobic core but also at the oil–water interface after binding to lipids. At the same time, the modification of inulin did not cause significant effects on the reducing ability of liposomes. Since the experiment was carried out when it was just made, this meant that the inulin was only in the water phase and could not penetrate into the oil–water interface, let alone the hydrophobic core.

The presence of secondary products (aldehydes, ketones, alkanes, organic acids and carbonyl groups) is a landmark product that should be monitored when monitoring the peroxide reaction [35]. The changes in TBARS were determined by detecting MDA, the product of the peroxidation. The influence of inulin modification on the formation of liposomes’ TBARS is shown in Figure 6C. The same situation with FRAP was observed in the TBARS assay. In general, inulin modification had little effect on the antioxidant properties of liposomes. At the same time, the content of MDA in the mixture was higher, which indicated that the encapsulated cinnamaldehyde had stronger antiperoxidative activity. Although the presence of inulin increased the rigidity of the liposomal bilayer membrane, the presence of inulin might also lead to instability by forming a dense region with phospholipids, inducing local dehydration of the bilayer membranes. When the inulin concentration was too high, the number of water-binding sites on the membrane increased, which would favor vesicle aggregation because it reduced the repulsive effect that prevents the two membranes from approaching, thereby limiting the antioxidant effect of cinnamaldehyde liposomes [45]. This result could partly explain the difference in the retention of cinnamaldehyde in the lipid bilayer under different inulin concentrations, because lipid oxidation would affect the permeability of the membrane, which in turn affected the leakage of cinnamaldehyde [24].

## 4. Conclusions

This study provides insights into the storage and thermal stability of inulin-modified cinnamaldehyde liposomes. The liposome was modified with inulin due to the hydrogen bond formation. This played a greater role in maintaining the stable state of the liposome. The inulin-modified liposomes still maintained good storage stability. Inulin-modified liposomes were more stable after pasteurization, with enhanced thermal stability and antioxidant effects. We reveal the distribution of inulin in lipid bilayers and its relationship with the liposome structure. Raman and FTIR spectra analysis showed that inulin can modulate the structural rigidity of liposome membranes, which largely depends on its concentration. Inulin concentrations were closely related to the stability of liposomes. The addition of 1.5% inulin enhanced the stability of liposomes most significantly. However, when the inulin was in excess, the capacity of the bilayer membrane to load cinnamaldehyde was reduced, the membrane permeability was enhanced and the stability of the system was reduced. So, the stability of liposomes first increased and then decreased with the increase in inulin concentration. The results showed that inulin can be used as an ideal choice for the modification and stability of food liposomes.

## Figures and Tables

**Table 1:** The order parameters and their change rates for the lateral interaction between chains of cinnamaldehyde liposomes with different inulin contents.

Inulin Content (%, *w*/*w*)	Blank Sample *	0	0.75	1.5	3	6	12
I_CH2_	1.4867	1.4701	1.7429	1.6423	1.5402	1.5792	1.8614
S_L_	0.5245	0.5134	0.6953	0.6282	0.5602	0.5861	0.7743
(S_L_ − S_L,A_)/S_L,A_	--	--	35.43%	22.37%	9.11%	14.17%	50.82%
I_C=C_	0.5493	1.7906	1.1299	1.6365	3.3313	1.9802	1.1009
S_T_	0.3104	1.0116	0.6384	0.9246	1.8821	1.1188	0.6220
(S_T_ − S_T,0_)/S_T,0_	--	--	−36.90%	−8.60%	86.04%	10.59%	−38.52%

* means the pure liposomes without cinnamaldehyde-loading.

## Data Availability

The data presented in this study are available on request from the corresponding author.
